# Empathy versus Parsimony in Understanding Post-Conflict Affiliation in Monkeys: Model and Empirical Data

**DOI:** 10.1371/journal.pone.0091262

**Published:** 2014-03-17

**Authors:** Ivan Puga-Gonzalez, Marina Butovskaya, Bernard Thierry, Charlotte Korinna Hemelrijk

**Affiliations:** 1 Behavioural Ecology and Self-organization, Centre for Ecological and Evolutionary Studies, University of Groningen, Groningen, The Netherlands; 2 Institute of Ethnology and Anthropology, Russian Academy of Sciences, Moscow, Russia; 3 Départment d'Ecologie, Physiologie & Ethologie, Institut Interdisciplinaire Hubert Curien, Centre National de la Recherche Scientifique, Université de Strasbourg, Strasbourg, France; Arizona State University, United States of America

## Abstract

Post-conflict affiliation between former opponents and bystanders occurs in several species of non-human primates. It is classified in four categories of which affiliation received by the former victim, ‘consolation’, has received most attention. The hypotheses of cognitive constraint and social constraint are inadequate to explain its occurrence. The cognitive constraint hypothesis is contradicted by recent evidence of ‘consolation’ in monkeys and the social constraint hypothesis lacks information why ‘consolation’ actually happens. Here, we combine a computational model and an empirical study to investigate the minimum cognitive requirements for post-conflict affiliation. In the individual-based model, individuals are steered by cognitively simple behavioural rules. Individuals group and when nearby each other they fight if they are likely to win, otherwise, they may groom, especially when anxious. We parameterize the model after empirical data of a tolerant species, the Tonkean macaque (*Macaca tonkeana)*. We find evidence for the four categories of post-conflict affiliation in the model and in the empirical data. We explain how in the model these patterns emerge from the combination of a weak hierarchy, social facilitation, risk-sensitive aggression, interactions with partners close-by and grooming as tension-reduction mechanism. We indicate how this may function as a new explanation for empirical data.

## Introduction

Cognitively complex explanations have been given for many aspects of social behaviour in primates. For instance, post-conflict affiliation between former opponents of a fight and bystanders are usually referred to as ‘consolation’ and ‘appeasement’. Whether the assumption of high cognition underlying such social behaviour is justified is unsure, but it appears difficult to find cognitively simpler explanations. Here we use a combination of a computer model ‘GrooFiWorld’ based on self-organisation [1] and empirical data of a tolerant species of macaques, Tonkean macaques (*Macaca tonkeana*) to investigate what mechanisms may underly the occurrence of four forms of post-conflict affiliation between former opponents of a fight and bystanders, namely ‘appeasement’, which is when the former aggressor receives affiliation, ‘consolation’ when the former victim receives it, ‘solicited appeasement’ when the former aggressor solicits affiliation from a bystander (i.e. it initiates affiliation), and ‘solicited consolation’ when the former victim solicits it. In order to avoid the use of such anthropomorphic terms, we will refer to these categories of interaction as post-conflict affiliation that is received or solicited by former aggressors and victims.

Several functions and underlying cognitive mechanisms have been suggested for these postconflict interactions: relieving stress, reducing the risks of redirected aggression, recruiting support, strengthening bonds with valuable partners (i.e. individuals with whom they groom the most), and substituting reconciliation [2]–[8]. As to the cognitive mechanisms, special emphasis has been put on the post-conflict affiliation directed to the victim, i.e. ‘consolation’. Consolation was found to occur in apes but not in monkeys. This result has been interpreted as indicating a constraint of the cognitive capacity of monkeys, i.e. the cognitive constraint hypothesis [9]. According to this hypothesis, ‘consolation’ happens if a bystander recognizes that the victim is in distress and tries to alleviate the victim's distress. The absence of ‘consolation’ in monkeys has been attributed to their lack of ‘cognitive empathy’ [10]. ‘Consolation’, however, has recently been found in dogs (*Canis familiaris*) [11], wolves (*C. lupus*) [12], horses (*Equus caballus*) [13], rooks (*Corvus frugilegus*) [14], and in two species of monkeys [15], [16], from which it is known that their cognitive abilities are less developed than those of apes. Thus, whether cognitive empathy is a prerequisite for the occurrence of ‘consolation’ is questionable. The social constraint hypothesis is more parsimonious. It states that instead of a difference in cognitive abilities, the occurrence of ‘consolation’ may be related to a difference in the risks of aggression in different societies when approaching a former opponent [9]. In species with a tolerant dominance style the risks of further aggression after a conflict are lower than in species with an intolerant dominance style, making such affiliation more likely. In line with this is the fact that the only monkey species in which consolation has been confirmed are species that are tolerant, namely the stump-tailed (*M. arctoides*) and Barbary macaques (*M. sylvanus*) [15]–[18]. This hypothesis, however, does not explain why such affiliative postconflict behaviour happens in the first place.

In the present study, we are interested in the minimal cognitive abilities required to generate these four categories of post-conflict interaction. To investigate this, we chose the individual-based model ‘GrooFiWorld’ because in it individuals lack the cognitive abilities thought necessary to display consolation, i.e. individuals lack cognitive empathy and the motivation to ‘console’. Instead, in the model individuals behave according to simple rules of thumb: they tend to group and when they are near another individual they fight if their chance of winning is high; if they decide not to fight, they consider grooming especially when they are anxious [1], [19]. Despite these cognitively simple rules, this model has already reproduced many complex behavioural patterns that resemble those of primates. For example, in the model individuals reciprocate both grooming and support and interchange grooming for support [19] even though they do not keep record of acts given and received as has been assumed to be necessary [20]. Moreover, individuals reconcile fights, especially with valuable partners, and do so more often in tolerant than in intolerant societies, despite the fact that they lack memory of former opponents and a conciliatory disposition [1].

We compare the results of the model to empirical data of a monkey species known for its high level of social tolerance, relaxed dominance relationships, and its great propensity for affiliative contacts and appeasement *i.e.* the Tonkean macaque (*Macaca tonkeana*) [21]–[24]. Furthermore, because several empirical studies have shown that individuals who groom each other more often also are more often involved in consolation [2], [8], [16], [25], [26], we also study the relation between consolation and ‘valuable partners’ in the model and empirical data.

Because of the high level of social tolerance in Tonkean macaques, we expect to find the four categories of post-conflict affiliation. Furthermore, we compare the frequency and distribution of post-conflict affiliation in the empirical data and the model. The behavioural mechanisms we use to explain these patterns in the model may hold for empirical data also.

## Results

### Tonkean macaques and the GrooFiWorld model

Among female Tonkean macaques and females in the GrooFiWorld model, all four categories of post-conflict affiliation were found (Table 1). According to the MC-PC method, in both the empirical study and the model, aggressors and victims received and solicited post-conflict affiliation at similar rates (Table 1) (for details on the calculation see collection and analysis of empirical data in methods and analysis of affiliative tendencies in text S1). Aggressors were higher in rank than victims when they solicited and received post-conflict affiliation (Mann-Whitney U-test: soliciting, empirical data: n_Agr_ = 27, n_Vct_ = 20, U = 391, p<0.01; model: n_Agr_ = 541, n_Vct_ = 539, U = 240671, p<0.001; receiving, empirical data: n_Agr_ =  28, n_Vct_ = 10, U = 195, p<0.07; model: n_Agr_ = 666, n_Vct_ = 656, U = 345941, p<0.001). Furthermore, in both empirical data and model, aggressors and victims received more post-conflict affiliation than they solicited, but this was significant only in the model and not in empirical data (aggressors: Wilcoxon matched-pairs for received affiliation vs solicited affiliation, in model: n = 14, U = 55, p = 0.002; in Tonkean macaques: n = 13, U = 41, p = 0.50. victims: Wilcoxon matched-pairs for received affiliation vs solicited affiliation, in model: n = 14, U = 55, p = 0.002; in Tonkean macaques: n = 10, U = 20, p = 0.83).

**Table 1:** Frequency of post-conflict affiliative tendencies between former opponents and bystanders in empirical data and the GrooFiWorld model.

	Received post-conflict affiliation from a bystander by		Wilcoxon paired test	Solicited post-conflict affiliation from a bystander by		Wilcoxon paired test
	Aggressor	Victim		Aggressor	Victim	
**A) Empirical Data**	12.0	11.7	n.s.	3.2	7.0	n.s.
**B) GrooFiWorld Model**	15.5	13.2	n.s.	3.5	5.4	n.s.

Results of the model are averaged over 10 runs.

**p<0.01,

***P<0.001,

n.s. = non significant.

### Causes of post-conflict affiliation in the model

To understand what causes these patterns in the model we investigated the consequences of four different manipulations in the model on post-conflict affiliation (Table 2). We 1) switched off social facilitation so that individuals located close to a fight are no longer more likely to be the ones who are activated next, 2) omitted the effects of proximity by making individuals interact with partners we chose at random, 3) switched off the increase of anxiety after a fight, and 4) made grooming independent of anxiety. For further details on the manipulations see methods and Text S1.

**Table 2:** Post-conflict affiliative tendencies after performing four different manipulations in GrooFiWorld (see methods).

	Receipt of post-conflict affiliation		Solicitation of post-conflict affiliation	
	Aggressor	Victim	Aggressor	Victim
1) GrooFiWorld (complete model)	15.5	13.2	3.5	5.4
Experiments in the model:				
2) No social facilitation	**0.0**	**0.0**	7.1	8.0
3) Interaction partners chosen at random	**0.0**	**−3.8**	13.7	13.7
4) No increase in anxiety after a fight	15.5	14.2	0.8	2.1
5) No Anxiety induced grooming	16.3	14.0	**0.0**	**0.0**

Tendencies that are 0 or negative are given in bold.

When social facilitation is switched off or when individuals choose interaction partners at random, in both cases, post-conflict affiliation is no longer received from by-standers (2–3 in table 2). This is because social facilitation induces individuals close to a fight (bystanders) to be activated next and thus, to interact with one of the former opponents. Consequently, bystanders groom former opponents sooner after a fight than during the matched-control period. In the case when interaction partners are chosen at random, former opponents no longer receive post-conflict affiliation because the likelihood that a ‘bystander’ grooms a former opponent during the post-conflict period is the same as in the matched control (3 in table 2).

When the increase of anxiety after a fight is switched off, the solicitation of post-conflict affiliation decreases (compare 1 and 4 in Table 2), and it completely disappears when grooming is independent of anxiety (5 in Table 2). Thus, solicitation of post-conflict affiliation depends on the anxiety level of the former opponent because this influences its tendency to groom.

### Social relationships in the model and empirical data

In empirical data of Tonkean macaques and in GrooFiWorld the four categories of post-conflict affiliation were more frequent among partners that groomed each other more often. The specific associations in aggressors (Table 3) and victims (Table 4) are 1) former opponents directed more post-conflict affiliation to those bystanders from whom they received more post-conflict affiliation, i.e. reciprocation, 2) former opponents solicited more frequently post-conflict affiliation from those bystanders to whom they directed more grooming, and 3) those former opponents that were involved in post-conflict affiliation with each other more frequently were also involved in grooming interactions with each other more often (1, 5, 7, 8 in Table 3 & 4). A number of correlations were significant only in GrooFiWorld: former opponents received more frequently post-conflict affiliation from those bystanders 1) from whom they received grooming more frequently, and 2) to whom they directed grooming more frequently, and 3) former opponents solicited more frequently post-conflict affiliation from those bystanders from whom they received grooming more frequently (2, 3, 6 in Table 3 & 4). In the empirical study, the data of victims were insufficient for the TauKr matrix correlations (Table 4).

**Table 3:** Social relationships and post-conflict interactions between aggressors and bystanders in Tonkean macaques and GrooFiWorld.

	GrooFiWorld	Emp. Data
**Aggressors received PC affiliation more frequently from those bystanders:**		
1) to whom they also directed PC affiliation more frequently after a conflict	**0.07** *	**0.42** **
2) from whom they also received grooming more frequently in other context	**0.17** **	0.09
3) to whom they also directed grooming more frequently	**0.18** **	0.05
**Aggressors solicited PC affiliation more frequently from those bystanders:**		
4) from whom they received PC solicitation more frequently after a conflict	‡ **0.06** *	−0.13
5) to whom they directed grooming more frequently in other context	**0.19** **	**0.26** *
6) from whom they received grooming more frequently	**0.19** **	0.07
**Aggressors involved more frequently in grooming:**		
7) Also received PC affiliation from each other more frequently	**0.26** **	**0.39** **
8) Also solicited PC affiliation from each other more frequently	**0.23** **	**0.69** ***

Matrix TauKr correlations:

*p<0.05,

**<0.01,

***p<0.001.

PC = post-conflict.

‡ 1 correlation (5% of 16) is considered to be a type I error.

**Table 4:** Social relationships and post-conflict interactions between victims and bystanders in Tonkean macaques and GrooFiWorld.

	GrooFiWorld	Emp. Data
**Victims received PC affiliation more frequently from those bystanders:**		
1) to whom they also directed PC affiliation more frequently after a conflict	**0.10** **	NA
2) from whom they also received grooming more frequently in other context	**0.17** **	NA
3) to whom they also directed grooming more frequently	**0.21** **	NA
**Victims solicited PC affiliation more frequently from those bystanders:**		
4) from whom they received PC solicitation more frequently after a conflict	0.03	NA
5) to whom they directed grooming more frequently in other context	**0.19** **	NA
6) from whom they received grooming more frequently	**0.17** **	NA
**Victims involved more frequently in grooming:**		
7) Also received PC affiliation from each other more frequently	**0.25** **	NA
8) Also solicited PC affiliation from each other more frequently	**0.21** **	NA

Matrix TauKr correlations:

*p<0.05,

**<0.01,

***p<0.001.

PC = post-conflict; NA = not available (correlations could not be performed due to few data points).

## Discussion

In the empirical data of Tonkean macaques and in the GrooFiWorld model, we found all the four categories of post-conflict affiliation between former opponents and bystanders. The frequency and distribution of post-conflict affiliation received and solicited appeared to be similar in the empirical data and the model: a) aggressors solicited and received affiliation at similar rates as victims, b) aggressors and victims received more post-conflict affiliation than they solicited, c) they received and solicited post-conflict affiliation more frequently from those bystanders with whom they had a strong grooming relationship, and d) they reciprocated post-conflict affiliation.

Our model suggests two mechanisms as causes for the emergence of these post-conflict affiliations: social facilitation and anxiety reduction. As regards affiliation received from bystanders (‘consolation’ and ‘appeasement’), the model suggests that social facilitation is the main mechanism driving it. In the model, social facilitation increases the chances of bystanders to be activated and thus, bystanders are more likely to interact with former combatants soon after the fight. As regards solicited post-conflict affiliation (‘solicited consolation’ and ‘solicited appeasement’), the model suggests that this may emerge when former combatants intend to relieve their own anxiety by grooming bystanders. Empirical evidence seems to support both mechanisms, i.e. social facilitation and anxiety reduction. Social facilitation has been suggested to mediate post-conflict affiliation received by former opponents in Barbary macaques [16]. As to the reduction of anxiety, in Tonkean macaques and hamadryas baboons (*Papio hamadryas*), the increase in the rate of affiliation among bystanders after a fight has been attributed to an elevation of social tension and anxiety [23], [27] and in Barbary macaques victims of aggression significantly reduce their anxiety (measured as self-scratching) through soliciting consolation [16].

In the empirical data and in the model aggressors solicited post-conflict affiliation at similar rates as victims did and received post-conflict affiliation also at similar rates as victims did. This is unexpected because aggressors were usually higher in rank than victims, and thus one would expect aggressors to direct less affiliation and receive more of affiliation than victims. The similarity of frequency in post-conflict affiliation between aggressor and victim was probably due to the shallowness of the dominance hierarchy. Consequently, bystanders perceive approximately the same risks when approaching dominant and subordinate individuals and thus, they groom both at similar rates during the post-conflict period.

Besides, in the model, former opponents (i.e. aggressors and victims) received more post-conflict affiliation than they solicited. This pattern emerges because after a conflict former opponents are less likely to be activated again and thus less likely to groom bystanders (i.e. solicit post-conflict affiliation). Similarly, in real monkeys, receiving post-conflict affiliation may be more frequent than soliciting because during the post-conflict period former opponents are still focused on their previous opponent rather than on bystanders.

Furthermore, in the model and in Tonkean macaques former opponents affiliated more with those bystanders with whom they had a stronger grooming relationship (Table 3 and 4). This is also found in several other primate species [16], [25], [26], [28], [29]. Note that when valuable partners provide post-conflict affiliation to the former opponent, this is usually interpreted as an expression of cognitive empathy [30]. In our model, however, this pattern emerges as side effect of the spatial structure of the group because individuals have a relatively stable spatial position which causes them to interact more with some partners than with others [1], [19], [31], [32]. In agreement with this the correlation between post-conflict affiliation and grooming frequency disappear when individuals interact with partners at random (Table S1 and S2). Note that in the model ‘reconciliation’ with valuable partners has emerged in a similar way: individuals are usually closer to those with whom they groom more (which are their valuable partners), and thus they groom them more often also after a fight, which is labelled as ‘reconciliation’ [1].

At present, all macaque species in which ‘consolation’ has been confirmed are socially tolerant [15], [16]. This is consistent with the social constraint hypothesis, which argues that individuals from tolerant species have a higher degree of freedom in their social relationships than those from intolerant species, meaning that in tolerant species individuals can approach each other more easily [33], [34]. Indeed also in the model, the frequency of post-conflict affiliation is significantly higher at low intensity of aggression than at high intensity of aggression. However, the explanation of the model differs from that of the social constraint hypothesis. It is identical to our earlier explanation why there is less reconciliation also at high than low intensity (Puga-Gonzalez et al. 2009). Namely, the frequency of post-conflict affiliation in the model is lower at high intensity than at low intensity as a side-effect of the lower relative frequency of grooming versus aggression at high intensity of aggression. The lower frequency of grooming versus aggression is a side-effect of the spatial centrality of dominants which is more pronounced at high than at low intensity of aggression. The spatial centrality causes dominants to meet others more often and thus interact with others more often than subordinate individuals do, because subordinates are more often located at the periphery of the group. The relative higher frequency of interactions by dominants at high intensity, cause a relatively lower frequency of grooming versus aggression.

The model GrooFiWorld proposes an integrative theory of affiliative and aggressive behaviour of primates. One of the key traits in the model is aggression. Aggression causes the spatial structure of the group [1], [19], [31], [32], [35], [36] which influences the distribution of affiliative behaviour resulting in patterns such as reciprocation of grooming and support, exchange of grooming for support and support for grooming, reconciliation, and reconciliation with valuable partners [1], [19]. When in the model intensity of aggression is high, many of the patterns that emerge resemble those found in intolerant societies: the dominance hierarchy is steep; individuals direct grooming up the dominance hierarchy and towards individuals of similar rank; aggression and opposition (i.e. attacking one of two opponents while intervening in their fight) [20] are unidirectional; conciliatory tendency, time spent grooming and fighting are low; and female dominance over males is high [1], [35], [37]. In addition, in the model individuals receive more opposition from those to whom they direct more grooming and direct more often opposition to those by whom they are groomed more frequently; these patterns are similar to empirical data of three intolerant species of macaques (unpublished data). Remarkably, all these behavioural patterns emerge without assuming sophisticated cognition. Instead, these patterns emerge from cognitively simple behavioural rules in combination with the spatial structure of the group. The model also suggests that patterns are interconnected and depend on the dominance style (tolerant or intolerant), which is in line with the covariation hypothesis which states that social traits associate in clusters through development and evolution [38]. The results obtained so far give us confidence that the model GrooFiWorld captures at least some essential traits of real primate societies, and it is useful as a null model for empirical studies.

## Methods

### Ethics statement

This study complied with French laws under the permission N°67-100 given by the French Agricultural Department. The group ranged semi-free in a wooded park of approximately one acre surrounded by fences, which included an indoor cage [39]. Monkey commercial diet and water were available *ad libitum*. Fresh food was distributed once a week but not during observations.

### Empirical

#### Subjects

The study was conducted on a well-established group of Tonkean macaques at the Primate Centre of Strasbourg, France. During the period of study, the group comprised 35 to 38 individuals, 19 adults (8 males and 11 females), 6 subadults (3 males and 3 females), 7 juveniles and 0–3 infants. Subadults were between 3 and 5 yrs old, and juveniles ranged between one and three years. All animals were present throughout the study. In the present paper we confine ourselves to females (n = 14; 11 adults and 3 subadults).

#### Collection and Analysis of Empirical Data

The study comprised 605 hours of observation. Aggressive behaviour included chasing, lunging, slapping, grabbing, biting or fierce biting; and non-aggressive behaviour included avoidance, lipsmack, screaming, or fleeing. We distinguished four different categories of post-conflict affiliation with affiliation received by former opponents from bystanders (i.e. ‘consolation’ and ‘appeasement’) and with affiliation solicited by former opponents from bystanders (i.e. ‘solicited consolation’ and ‘solicited appeasement’). Post-conflict affiliation behaviour was recorded following de Waal & Yoshihara [40]. After an agonistic interaction, either the victim or aggressor was followed during a 5-min post-conflict period (PC). PCs were restarted if aggression recurred within 30 s after the beginning of the PC. A 5-min matched-control period (MC) of the focal individual was taken on the next possible observation day at approximately the same time. Affiliative interactions comprised: sitting in contact, allogrooming, social play, mount, embrace, gentle touch, lipsmack and bared-teeth display [41]. To compare PC and MC, we divided the periods into blocks of 10 seconds (10-s block) and recorded the block in which the first affiliative contact between former opponents occurred. PC-MC pairs were called ‘attracted’ when the affiliative contact occurred earlier in PC period than in MC period; ‘dispersed’ when it occurred earlier in MC than in PC; and ‘neutral’ when it occurred during the same 10-s block in MC and PC period or when no contact occurred in either PC or MC period [40]. To calculate the affiliative tendency we used the improved formula for measuring conciliatory tendency: number of attracted pairs minus dispersed pairs divided by the total number of pairs (Equation 1) (for an example of the calculation see text S1) [42]. A total of 251 PC-MC pairs were collected, that consisted of 168 and 83 PC-MC pairs for aggressors and victims respectively (outdoor and indoor cases were merged into one sample). We compare the rank of aggressors with those of the victims by means of the Mann-Whitney U-test. The dominance rank of each individual was calculated based on the average dominance index [43].
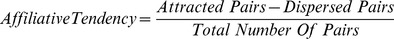
(1)


#### Matrix correlations

We used matrix TauKr correlations [44] to test for reciprocity of post-conflict affiliation and to test whether former opponents solicited or received more affiliation from those bystanders: 1) from whom they also received grooming more frequently; 2) to whom they also directed grooming more frequently; 3) with whom they were also involved in grooming more frequently. For further details see text S1.

### Modelling

#### The model GrooFiWorld

A full description of the model can be found in text S1 and in our earlier papers [1], [19]. Here we only present a summary. In the model, individuals tend to group, compete and affiliate. The effects of winning and losing a fight are self-reinforcing [35], [36], [45]–[48]. When the risk of losing a fight is high, individuals tend to avoid it and may groom instead. The decision whether to groom or not depends on their degree of anxiety: the more anxious, the more inclined to groom. As indicated by empirical studies, individuals: 1) become more anxious after a fight [49]–[51]; 2) reduce their anxiety when receiving affiliative behaviour (i.e. grooming) and to a lesser degree when actively grooming [51]–[53]; and 3) increase their motivation to groom when they have not been receiving grooming for some time, and decrease their motivation to groom after giving or receiving grooming [54]–[56]. Individuals are activated in random order; however, when an individual is spatially close to a fight (i.e. within the radius of social facilitation, see Table S3) then its chances of being activated earlier increase. Below, we describe the parameters in the model, and the way data were collected and analysed, post-conflict affiliation tendencies were measured, and experiments were done.

#### Parameters

We kept most of the parameter values the same as in our previous studies (Table S3) [1], [19], [35], [36] and tuned other parameters of the model to those of empirical data regarding group size (25 individuals), sex ratio (14 females, 11 males), intensity of aggression (a low value), relative frequency of grooming and aggression (4∶1), female dominance (0.48) [37], and the distribution of dominance values [43]. The distribution of the dominance values we calculated by filling in dominance values between the highest and lowest dominance in the model using dominance indices from empirical data [35], [43]. For results of the model with different ratios of the frequency of grooming versus fighting see table S4; for more details, see text S1.

#### Experiments in the Model

To understand what caused the patterns of post-conflict affiliation in the model, we manipulated it in four different ways. First, we switched off ‘social facilitation’. Social facilitation implies that an individual located close to a fight increases its likelihood of being activated next (for details see text S1). Second, we investigated the role of interactions being based on proximity by making individuals interact with partners chosen at random. Third, we switched off the increase of anxiety after a fight in the former opponents. Fourth, we made grooming independent of anxiety; thus, when individuals decided that it was too risky to fight, we made them decide by chance whether or not to groom their partners. In all experiments, the average number of interactions per individual and the relative frequency of grooming and fighting were kept the same as in the complete model.

#### Data collection

Every run consisted of 350 periods and each period consisted of 500 activations (i.e. group size (n = 25) multiplied by 20). Data were collected from period 200 to 350 to exclude any bias caused by transient values. For each activation, we recorded the spatial position and heading of each individual. With respect to fighting we recorded the identity of the winner and loser and with respect to grooming that of the actor and receiver. We ran 10 independent replicas. The results are shown as the average value of the statistic over 10 runs. Their combined probability is based on the improved Bonferroni procedure [57]. We used non-parametric statistics and two-tailed probabilities; however, if patterns were predicted by empirical studies, we used one-tailed probabilities.

#### Analysis of affiliative tendencies

We analysed the four different categories of post-conflict affiliation between former opponents and bystanders by means of the PC-MC method in the same way as has been done empirically [40]. We focused exclusively on females (n = 14). As in our previous analysis of reconciliation, the length of PC and MC periods was set to the next three activations of the focal opponent after the start of the MC or PC [1]. One day was represented by one period of the model, i.e. 500 activations. PCs were restarted if aggression recurred within the first activation of the former opponents after the start of the PC. PC-MC pairs were classified as ‘attracted’, ‘dispersed’, and ‘neutral’ (see above for a definition). For an example of the calculation see text S1.

To analyse associations between Post-conflict affiliation and grooming among group members, we performed the same matrix correlations with the data of the model as we did with empirical data (see section on Matrix correlations and text S1).

## Supporting Information

Table S1 **The effect of random interactions among individuals (instead of interactions based on proximity) on social relationships and post-conflict interactions between aggressors and bystanders in GrooFiWorld.** Matrix TauKr correlations. The values of the coefficients are the average of ten runs. PC = post-conflict.
(DOCX)

Table S2 **The effect of random interactions among individuals (instead of interactions based on proximity) on social relationships and post-conflict interactions between victims and bystanders in GrooFiWorld.** Matrix TauKr correlations. The values of the coefficients are the average of ten runs. PC = post-conflict.
(DOCX)

Table S3 **Value of parameters in the model ‘GrooFiWorld’.**
(DOCX)

Table S4 **Effects of different ratios of the frequency of grooming versus fighting (groom : fight) on frequency of post-conflict affiliative behaviour with bystanders in the model GrooFiWorld.** Results of the model are averaged over 10 runs. For comparison results of the empirical data are also shown.
(DOCX)

Text S1 **Supporting information on the methods.**
(DOCX)
