# Supplementary material for: Empathy versus Parsimony in Understanding Post-Conflict Affiliation in Monkeys: Model and Empirical Data
Source: PLoS One. 2014 Mar 17;9(3):e91262. doi: 10.1371/journal.pone.0091262 (PMC3956673; doi:10.1371/journal.pone.0091262)
Supplement: Table S2 — The effect of random interactions among individuals (instead of interactions based on proximity) on social relationships and post-conflict interactions between victims and bystanders in GrooFiWorld. Matrix TauKr correlations. The values of the coefficients are the average of ten runs. PC = post-conflict. (DOCX) [file pone.0091262.s002.docx]

**Table S2. The effect of random interactions among individuals (instead of interactions based on proximity) on social relationships and post-conflict interactions between victims and bystanders in GrooFiWorld.**

| GrooFiWorld with random interactions | TauKr coefficient | |
| --- | --- | --- |
| **Victims received PC affiliations more frequently from those bystanders:** | | |
| 1) to whom they directed PC affiliations more frequently after a conflict | 0.01 | |
| 2) from whom they received grooming more frequently also in other context | 0.00 | |
| 3) to whom they also directed grooming more frequently | -0.01 | |
| **Victims solicited PC affiliations more frequently from those bystanders:** | | |
| 4) from whom they received PC solicitations more frequently after a conflict | 0.00 | |
| 5) to whom they directed grooming more frequently in other contexts | 0.01 | |
| 6) from whom they received grooming more frequently | 0.00 | |
| **Victims involved more frequently with each other in grooming:** |  |  |
| 7) also received PC affiliations from each other more frequently | 0.02 | |
| 8) also solicited PC affiliations from each other more frequently | 0.01 | |

Matrix TauKr correlations. The values of the coefficients are the average of ten runs. PC= post-conflict.
