# Supplementary material for: Empathy versus Parsimony in Understanding Post-Conflict Affiliation in Monkeys: Model and Empirical Data
Source: PLoS One. 2014 Mar 17;9(3):e91262. doi: 10.1371/journal.pone.0091262 (PMC3956673; doi:10.1371/journal.pone.0091262)
Supplement: Table S3 — Value of parameters in the model ‘GrooFiWorld’. (DOCX) [file pone.0091262.s003.docx]

**Table S3. Value of parameters in the model ‘GrooFiWorld’.**

| Parameter | Description | Females | Males |
| --- | --- | --- | --- |
| General Parameters |  |  |  |
| GroupSize | Total number of individuals | 25 |  |
| Sex ratio | Number of | 14 | 11 |
| Female Dominance | Relative position of females over males in the dominance hierarchy [37]. | 0.48 | |
| InitRadius | Predefined space at start of simulation | 1.7*# Inds | 1.7*# Inds |
| Radius of social facilitation | Radius starting from centre point between two opponents | 10 | 10 |
| **Grouping Parameters** |  |  |  |
| PersSpace | Close encounter distance | 8 | 8 |
| NearView | Medium distance | 24 | 24 |
| MaxView | Maximal viewing distance | 50 | 50 |
| SearchAngle | Turning angle to find others | 90° | 90° |
| VisionAngle | Angle of field of view | 90° | 90° |
| **Fighting Parameters** |  |  |  |
| InitDom | Initial Dom value | see main text ‘parameters in the model’ | |
| RiskAvers | Number of ‘mental battles’ | 2.75 | 2.75 |
| StepDom | Scaling factor for aggression intensity | 0.08 | 0.1 |
| FleeingDist | After losing a fight | 4.5 | 4.5 |
| ChaseDistance | After winning a fight | 2.5 | 2.5 |
| MoveAfterGroom | After grooming | 0.5 | 0.5 |
| **Grooming Parameters** |  |  |  |
| InitAnx | Initial anxiety value | 0.5 | 0.5 |
| AnxInc | Increase in anxiety after every activation | 1.2% | 1.2% |
| AnxDcrGree | Decrease of anxiety of groomee | 0.15 | 0.15 |
| AnxDcrGrmr | Decrease of anxiety of groomer | 0.1 | 0.1 |
| AnxIncFight | Increase of anxiety after fighting | 0.1 | 0.1 |
