# Supplementary material for: Empathy versus Parsimony in Understanding Post-Conflict Affiliation in Monkeys: Model and Empirical Data
Source: PLoS One. 2014 Mar 17;9(3):e91262. doi: 10.1371/journal.pone.0091262 (PMC3956673; doi:10.1371/journal.pone.0091262)
Supplement: Table S4 — Effects of different ratios of the frequency of grooming versus fighting (groom : fight) on frequency of post-conflict affiliative behaviour with bystanders in the model GrooFiWorld. Results of the model are averaged over 10 runs. For comparison results of the empirical data are also shown. (DOCX) [file pone.0091262.s004.docx]

**Table S4. Effects of different ratios of the frequency of grooming versus fighting (groom : fight) on frequency of post-conflict affiliative behaviour with bystanders in the model GrooFiWorld*.***

| Groom : Fight | Receipt of post-conflict affiliation | | Solicitation of post-conflict affiliation | |
| --- | --- | --- | --- | --- |
|  | Aggressor | Victim | Aggressor | Victim |
| 5:1 | 18.00 | 15.2 | 0.7 | 2.1 |
| 4:1 | 15.5 | 13.2 | 3.5 | 5.4 |
| 3:1 | 8.6 | 9.4 | 6.0 | 5.2 |
| 2:1 | 7.2 | 7.0 | 6.9 | 5.2 |
| 1:1 | 1.8 | 2.7 | 4.9 | 6.8 |
| Empirical Data | 12.0 | 11.7 | 3.2 | 7.0 |

Results of the model are averaged over 10 runs. For comparison results of the empirical data are also shown.
