# Supplementary material for: Empathy versus Parsimony in Understanding Post-Conflict Affiliation in Monkeys: Model and Empirical Data
Source: PLoS One. 2014 Mar 17;9(3):e91262. doi: 10.1371/journal.pone.0091262 (PMC3956673; doi:10.1371/journal.pone.0091262)
Supplement: Text S1 — Supporting information on the methods. (DOCX) [file pone.0091262.s005.docx]

**Methods**

## Empirical study

This study complied with French laws under the permission N°67-100 given by the French Agricultural Department. The group was ranging semi-free in a wooded park of approximately one acre surrounded by fences, which included an indoor cage [1]. Monkey commercial diet and water were available *ad libitum*. Fresh food was distributed once a week but not during observations.

*Collection and Analysis of Empirical Data*

Observations were conducted by the second author between 10 a.m. and 3 p.m. from September 15^th^ to December 15^th^ 1999 (330 hours), and from 1^st^ October to December 15^th^ 2000 (275 hours). Data were collected by all-occurrence sampling [2][Altmann, 1974](http://www.sciencedirect.com/science/article/pii/S0003347210001466#bbib2) J. Altmann, Observational study of behavior: sampling methods. *Behaviour*, **49** (1974), pp. 227–265.. Aggressors were followed during 1999 and victims during 2000. Agonistic interactions were recorded when an individual displayed aggressive behaviour to another and the receiver responded with aggressive or non-aggressive behaviour. A conflict was considered to be decided when one of the opponents unilaterally withdrew or submitted, and to be undecided when no clear signs of submission were shown by either opponent (e.g. counter-aggression). In case of polyadic conflicts, only the two initial combatants were considered for analysis. A total of 251 PC-MC pairs were collected: 213 outdoors (in the park) and 38 indoors (in a cage). Post-conflict affiliation concerned in 168 cases (outdoors and indoors) the aggressor and 83 cases (outdoors only) the victim. 14 females participated in aggressive encounters: 2 exclusively as aggressors (2 adults), 11 as aggressors and victims (8 adults and 3 subadults), and 1 as a victim exclusively (1 subadult).

*Matrix Correlations*

Sociometric matrices were built with data of interactions of approach and avoidance, grooming and post-conflict affiliation. Correlations between matrices were TauKr correlations and the level of significance was based on 2000 permutations [3,4]. Due to the high number of correlations, significant results may arise by chance. We corrected for this by discarding the 5% of correlations with the lowest significances (Type I error) per table of results. Matrices of post-conflict affiliative tendencies were corrected for opportunity, i.e. number of fights in which individuals were involved and thus could have affiliated with bystanders. Matrices of grooming given and received excluded the cases of grooming considered as a post-conflict affiliation.

*Analysis of affiliative tendencies*

To analyse the affiliative tendencies we used the MC-PC method [5]. After each fight, we started the post-conflict (PC) period whose length was set to 10 min for empirical data and the next three activations of the focal opponent for the model. During the PC period we recorded the type of affiliation that happened first and this was the type of affiliation we controlled for during the MC period. For instance, if during the PC an affiliative interaction initiated by a bystander was the first to happen, during the MC we only controlled for this type of affiliation. If during the MC there was no such interaction or if the affiliation happened first in the PC than in the MC, this would be counted as an 'attracted' pair for affiliation initiated by a bystander. Note, however, that if during the MC affiliative behaviour was instead initiated by the former opponent towards a bystander, this was not counted as a dispersed pair in the affiliation initiated by former opponent to avoid double counting data. Also, if no affiliation happened during the PC, we only considered the type of affiliation that happened first during the MC (i.e. affiliation initiated by bystander or by former opponent) and labeled this pair as ‘dispersed’.

## Modeling study

*The model*

We use an individual-based model called GrooFiWorld [6]. This is an extension of a previous model called Dom-World in which individuals group and compete [7-9]. The extension consists of grooming behaviour and that is why it is called ‘GrooFiWorld’ (i.e. Grooming and Fighting). The space of the ‘world’ is continuous, i.e. individuals are free to move in any direction. They have a certain angle of vision (*VisionAngle*) and a maximum distance of perception (*MaxView*). Individuals are provided with three behavioural tendencies: 1) to group, 2) to perform dominance interactions and 3) to display affiliative behaviour. At the start of each run, the individuals occupy random locations within a predefined circumference, *InitRadius*, the value of which is the product of a number and the number of individuals (Table S3). The individuals’ activities are regulated by a timing regime in which each individual receives a random waiting time from a uniform distribution and the individual with the shortest waiting time is activated first. This regime is combined with a biologically plausible timing regime reflecting a kind of ‘social facilitation’ (e.g. see Galef 1988) in which the waiting time of an individual is shortened when a dominance interaction occurs close by (radius of social facilitation, Table S3). This function was implemented after the observation that in social species specific events, such as a fighting, may produce an increase in arousal of bystanders (or observers) leading to a response [10]. For instance, De Marco et al. [11] have shown that after a fight, Tonkean macaque bystanders were more likely to increase affiliation, i.e. they became more active.

*Setting Parameters in the model*

Where possible we kept the same parameter values as in our previous studies (Table S3) [6-8,12]. However, to effectively reflect the social behaviour of the group of Tonkean macaques, we adjusted the settings of the model to the same group size, sex ratio, intensity of aggression, relative frequency of grooming and aggression, female dominance [13], and steepness of the hierarchy [14]. Thus, group size was set to n=25 individuals, 14 females and 11 males. Intensity of aggression was set to low values because, among Tonkean macaques, aggression is mild, i.e. mostly consisting of threats and slaps [15]. The relative frequency of grooming versus aggression in the group of Tonkean macaques was 10:1, which underestimates the frequency of aggression, because threats were not included. Since in the model we include also threats, we needed a lower ratio of grooming to aggression than the empirical one. To find a biologically relevant ratio, we investigated the effects of different ratios of grooming versus aggression on the frequency of post-conflict affiliative behaviour with bystanders (Table S4). This investigation showed that the frequency of post-conflict affiliative interactions increased with the relative frequency of grooming versus aggression (Table S4) and resembled empirical data most at a ratio of grooming to fighting of 4:1. Thus, we chose this ratio for our study. To adjust the relative frequency of grooming to aggression, the parameter values of increasing of anxiety (*AnxInc)* and of aversion to risk (*RiskAvers)* were increased (Table S3). After these modifications, the average distance among group members decreased dramatically from 20 to 4 units. Thus, we increased the distance over which an individual flees after losing a fight (*FleeingDistance),* the distance over which the winner of a fight chases its opponent (*ChaseDistance),* and the moving distance after grooming (*MoveAfterGroom*) (Table S3). In order to mimic the same female dominance and steepness of the hierarchy as in the empirical date, we first calculated the dominance index of each individual in our group of Tonkean macaques with the help of the matrix tester program [4,14]. The dominance index ranged from 0 to 1, with 1 being most dominant and 0 least dominant. Next, from a simulation of the model at low intensity of aggression, we took the minimum and maximum dominance values and we distributed the empirical dominance values within this range. The individual with the maximum dominance value in the model represented the individual of the empirical data with a dominance index of 1 and the individual with the minimum dominance value represented the macaque with a dominance index of 0. The other individuals in the model represented those macaques ranking in between these two extremes. The dominance values of these individuals were calculated based on the dominance index obtained from empirical data. For instance, in the model the individual representing a macaque with a dominance index of 0.5 got the average dominance value between the maximum and minimum. The dominance values were fixed during the whole simulation. This procedure implied automatically the same degree of female dominance relative to males and hierarchical gradient as in the empirical data. The data obtained from these simulations were used for the analyses of affiliative tendencies.

*Behavioural rules in the model*

Rules for grouping

Whenever an individual does not see another one close by (within its personal space, *PersSpace*), grouping rules come into effect. The individual starts looking for others at greater and greater distances (*NearView* and *MaxView*). If, even then, no one else is in sight, it turns over a *SearchAngle* in order to find others. In this way individuals tend to remain in a group. If, however, an individual spots another one close by, within its personal space (*PersSpace*), a social interaction may take place.

Rules for social interactions

Upon meeting someone else in its personal space (*PersSpace*), an individual first considers whether or not to perform a dominance interaction. This decision depends on the risks involved, whereby risk concerns the chance of losing a fight [8]. A fight is only initiated when the individual expects to win; if defeat is expected, it considers grooming the other. This decision depends on its degree of anxiety: the more anxious an individual is, the more inclined it is to groom.

Aggressive interactions

Dominance interactions are modeled as before [7] and are an extension of the DoDom rules of Hogeweg [16]. A dominance interaction takes place only if an individual expects to be victorious, i.e. it avoids risks. The risks of losing a fight are estimated by means of a ‘mental battle’, i.e. a representation of a real fight. During a ‘mental battle’ an individual *i* compares its dominance value (*Dom_i_*) relatively to that of his opponent *j* (*Dom_j_*) to a random value drawn from a uniform distribution between zero and one (Equation 1). This process is repeated for *RiskAvers* times (Table S3).

$w_{i}=\left[\begin{matrix} 1 \frac{DOM_{i}}{DOM_{i}+DOM_{j}}>RND(0,1) \\ 0 else \end{matrix}\right.$ (1)

If in all these cases, individual *i* expects to be victorious, then a real dominance interaction occurs. The outcome of the real dominance interaction is again decided according to equation 1.

To reflect the self-reinforcing effects of victory and defeat [13,17-19], dominance values are updated by increasing the dominance value of the winner and decreasing that of the loser by the same amount (Equation 2). This positive feedback is ‘damped’ because a victory from a higher ranking opponent increases its relative Dom-value only slightly, whereas an (unexpected) success from a lower ranking individual increases its dominance value by a greater amount. This ‘damped’ feedback, thus, allows for dominance reversals. To keep Dom-values positive, their minimum value is, arbitrarily, set at 0.01.

$\begin{matrix} DOM_{i}=DOM_{i}+\left(w_{i}-\frac{DOM_{i}}{DOM_{i}+DOM_{j}}\right)*STEPDOM \\ DOM_{j}=DOM_{j}-\left(w_{i}-\frac{DOM_{j}}{DOM_{i}+DOM_{j}}\right)*STEPDOM \end{matrix}$ (2)

The change in Dom-values is multiplied by a scaling factor, which represents the intensity of aggression (*StepDom*, see table S3) [7,20]. High values of *StepDom* indicate fierce aggression and cause a big change in Dom-values after a fight. Low values of *StepDom* represent mild aggression and have a lower impact in Dom-values.

After winning an interaction, the winner chases its opponent over a predefined *ChaseDistance* whereby the loser flees over a predefined *FleeingDistance* (Table S3). Both individuals subsequently turn randomly 45 degrees to right or left.

Grooming interactions

In our model, active and passive grooming reduce anxiety as indicated by empirical studies [21-25,25,26]. Furthermore, individuals increase their motivation to groom after not being groomed for some time and decrease their motivation after giving or receiving grooming as demonstrated in empirical studies [27-32].

In the model, therefore, grooming is induced by the level of *Anxiety* of the individual, which ranges from very relaxed to very tense, i.e. from 0 to 1. At the beginning of the simulation, all individuals start with the same *Anxiety* value, i.e. 0.5, *InitAnx* (Table S3). When an individual decides it is too risky to attack other, the individual may groom its partner if its level of *Anxiety* is higher than a random number; otherwise, it displays ‘non-aggressive’ proximity. Grooming reduces *Anxiety* (and thus the tendency to groom) in both, the groomee and the groomer. This reduction is stronger in the groomee (*AnxDcrGree*) than in the groomer (*AnxDcrGrmr*) (Table S3). After grooming, both partners turn over a small angle (45◦) randomly to the right or left and move over a predefined distance (*MoveAfterGroom,* Table S3) to avoid repeated interactions with the same partner. During periods without grooming, *Anxiety* increases. This increase is updated after every activation with *AnxInc* (Table S3). In addition, inspired by the observation that anxiety increases after a fight in real non-human primates and humans [33,34], *Anxiety* increases with *AnxIncFght* in both opponents after a fight in the model (Table S3).

**References**

References

1. Thierry B. (1985) Patterns of agonistic interactions in three species of macaque (*Macaca mulatta, M. fascicularis, M. tonkeana)*. Aggressive Behavior 11: 223-233.

2. Altmann J. (1974) Observational study of behaviour: Sampling methods. Behaviour 49: 227-267.

3. Hemelrijk CK. (1990) A matrix partial correlation test used in investigations of reciprocity and other social interaction patterns at group level. J. theor. Biol. 143: 405-420.

4. Hemelrijk CK. (1990) Models of, and tests for, reciprocity, unidirectional and other social interaction patterns at a group level. Anim Behav 39: 1013-1029.

5. Veenema HC, Das M, Aureli F. (1994) Methodological improvements for the study of reconciliation. Behavioural Processes 31: 29-38.

6. Puga-Gonzalez I, Hildenbrandt H, Hemelrijk CK. (2009) Emergent patterns of social affiliation in primates, a model. Plos Computational Biology 5: e1000630. doi:10.1371/journal.pcbi.1000630.

7. Hemelrijk CK. (1999) An individual-oriented model on the emergence of despotic and egalitarian societies. P Roy Soc Lond B Bio 266: 361-369.

8. Hemelrijk CK. (2000) Towards the integration of social dominance and spatial structure. Anim Behav 59: 1035-1048.

9. Hemelrijk CK. (2005) A process-oriented approach to the social behaviour of primates. In: Hemelrijk CK, editor. Self-organisation and evolution of social systems. Cambridge, UK: Cambridge University Press. pp. 81-107.

10. Galef BG,Jr. (1988) Imitation in animals: History, definitions, and interpretation of data from the psychological laboratory. In: Zentall T, Galef B, editors. Social learning: Psychobiological and biological perspectives. Hillsdale, New Jersey: Erlbaum. pp. 3-28.

11. De Marco A, Cozzolino R, Dessi-Fulgheri F, Thierry B. (2010) Conflicts induce affiliative interactions among bystanders in a tolerant species of macaque (*Macaca tonkeana*). Anim Behav 80: 197-203.

12. Hemelrijk CK, Puga-Gonzalez I. (2012) An individual-oriented model on the emergence of support in fights, its reciprocation and exchange. PLoS ONE 7: e37271.

13. Hemelrijk CK, Wantia J, Isler K. (2008) Female dominance over males in primates: Self-organisation and sexual dimorphism. PLoS ONE 3: e2678.

14. Hemelrijk CK, Wantia J, Gygax L. (2005) The construction of dominance order: Comparing performance of five different methods using an individual-based model. Behaviour 142: 1043-1064.

15. Thierry B. (2004) Social epigenesis. In: Thierry B, Singh M, Kaumanns W, editors. Macaque societies: A model for the study of social organisation. Cambridge: Cambridge University Press. pp. 267-289.

16. Hogeweg P. (1988) MIRROR beyond MIRROR, puddles of LIFE. In: Langton C, editor. Artificial life, SFI studies in the sciences of complexity. Redwood City, California: Adisson-Wesley Publishing Company. pp. 297-316.

17. Hsu Y, Wolf LL. (1999) The winner and loser effect: Integrating multiple experiences. Animal Behaviour 57: 903-910.

18. Setchell JM, Smith T, Wickings EJ, Knapp LA. (2008) Social correlates of testosterone and ornamentation in male mandrills. Horm Behav 54: 365-372.

19. Barchas PR, Mendoza SD. (1984) Emergent hierarchical relationships in rhesus macaques: An application of chase's model. In: Barchas PR, editor. Social hierarchies: Essays Towards a Sociophysiological Perspective. Westport, CT: Greenwood Press. pp. 81-95.

20. Hemelrijk CK. (1998) Risk sensitive and ambiguity reducing dominance interactions in a virtual laboratory. : 255-262.

21. Aureli F, Preston SD, de Waal FBM. (1999) Heart rate responses to social interactions in free-moving rhesus macaques (*Macaca mulatta*): A pilot study. J Comp Psychol 113: 59-65.

22. Aureli F, van Schaik CP. (1991) Postconflict behaviour in long-tailed macaques (*Macaca fascicularis*): II. coping with the uncertainty. Ethology 89: 101-114.

23. Castles DL, Whiten A. (1998) Post-conflict behaviour of wild olive baboons. II. stress and self-directed behaviour. Ethology 104: 148-160.

24. Das M, Penke Z, van Hooff JARAM. (1998) Postconflict affiliation and stress-related behaviour of long-tailed macaque aggressors. Int J Primatol 19: 53-71.

25. Shutt K, MacLarnon A, Heistermann M, Semple S. (2007) Grooming in Barbary macaques: Better to give than to receive? Biol Lett 3: 231-233.

26. Schino G, Scucchi S, Maestripieri D, Turillazzi PG. (1988) Allogrooming as a tension-reduction mechanism: A behavioral approach. American Journal of Primatology 16: 43-50.

27. Martel FL, Nevison CM, Simpson MJA, Keverne EB. (1995) Effects of opioid receptor blockade on the social behavior of rhesus monkeys living in large family groups. Dev Psychobiol 28: 71-84.

28. Fabre-Nys C, Meller RE, Keverne EB. (1982) Opiate antagonists stimulate affiliative behavior in monkeys. Pharmacol Biochem Be 16: 653-659.

29. Graves FC, Wallen K, Maestripieri D. (2002) Opioids and attachment in rhesus macaque (*Macaca mulatta*) abusive mothers. Behav Neurosci 116: 489-493.

30. Schino G, Troisi A. (1992) Opiate receptor blockade in juvenile macaques: Effect on affiliative interactions with their mothers and group companions. Brain Res 576: 125-130.

31. Meller RE, Keverne EB, Herbert J. (1980) Behavioral and endocrine effects of naltrexone in male talapoin monkeys. Pharmacol Biochem Be 13: 663-672.

32. Keverne EB, Martensz ND, Tuite B. (1989) Beta-endorphin concentrations in cerebrospinal-fluid of monkeys are influenced by grooming relationships. Psychoneuroendocrino 14: 155-161.

33. Aureli F, Cords M, Van Schaik CP. (2002) Conflict resolution following aggression in gregarious animals: A predictive framework. Anim Behav 64: 325-343.

34. Butovskaya ML. (2008) Reconciliation, dominance and cortisol levels in children and adolescents (7–15-year-old boys). Behaviour 145: 1557-1576.
